# Prevalence of Out-Of-Home Care Among School-Aged Children in Canada, 2002–2018: An Analysis of Nationally-Representative Student Survey Data

**DOI:** 10.3389/ijph.2025.1608481

**Published:** 2025-11-18

**Authors:** Prateek Sharma, Nathaniel J. Pollock, Wendy Hovdestad, Gabriela Williams, Lil Tonmyr

**Affiliations:** Centre for Surveillance and Applied Research, Public Health Agency of Canada, Ottawa, ON, Canada

**Keywords:** adolescents, alternative care, child welfare, child protection, public health surveillance

## Abstract

**Objectives:**

The objectives were to estimate the prevalence of out-of-home care among school-aged children in Canada by year, gender, age group, and placement type and assess time trends.

**Methods:**

We analyzed data from five cycles of the Health Behaviour in School-aged Children survey. Respondents were students in grades six through ten attending public schools in Canada. Based on a question about the family structure, we derived three types of living arrangements – (1) foster/children’s home, (2) kinship home, or (3) living with a parent(s) – and estimated the prevalence of each type.

**Results:**

The pooled sample included 93,720 students; 1.1% reported living in a foster/children’s home and 2.1% in a kinship home and in 2018. At the p = 0.05 level (chi-square), there were no observed differences in prevalence by gender or age group. Over time, the prevalence of living in a kinship home increased more than foster/children’s home (average percent change per cycle of 18.5% versus 5.0%), to 2.9% and 1.1%, respectively, in 2018.

**Conclusions:**

The prevalence of out-of-home care in Canada was higher that previous estimates based on census and administrative data.

## Introduction

Children and youth in out-of-home care experience elevated risks for social, physical, and mental health challenges, often due to early life exposure to maltreatment and poverty [1–3]. While such risks are recognized, national data in Canada about this subpopulation of young people involved in the child welfare system are limited [4–6].

Out-of-home care or “alternative care” [4] typically refers to children and youth who live with extended family, in a foster home, or in congregate or institutional setting like a group home [5]. The two primary sources of national point estimates of out-of-home care are the Census of Population (Census) and the Canadian Child Welfare Information System (CCWIS). The Census is a self-reported national household survey; since 2011, it has captured information about foster children who reside in private households [7]. Based on 2021 census data, an estimated 0.46% (n = 27,445) of children aged 0–14 were in a foster home [7]. The CCWIS is an administrative database that includes point-in-time count data about children and youth in out-of-home care on March 31 of each year [5]. A recent analysis of CCWIS data estimated that 0.84% of children in Canada (n = 61,104) were in out-of-home care on March 31, 2022 [5].

Although both the Census and CCWIS can be used to estimate the prevalence of out-of-home care in Canada, discrepancies between their estimates are expected. Each source uses a different definition of the population of interest, a different sampling frame, and a different data collection method [5]. Additionally, the coverage in each data source varies by geography, age, and placement type. An approach to offsetting design biases, coverage limitations, and data quality issues in these sources is to look for broad epidemiological patterns. This form of triangulation is a way to corroborate findings from diverse data sources [8] and inform data-driven decision-making.

Since 2002, the Health Behaviour in School-aged Children (HBSC) study included a question about family structure which makes it possible to distinguish between children living in homes with and without their parents [9]. This distinction has been used in to examine the extent to which children live in out-of-home care in previous international comparisons [10, 11]. HBSC data therefore provide an opportunity to assess trends in out-of-home care in Canada.

The objectives of this analysis were to: 1) estimate the prevalence of out-of-home care among school-aged children in grades six to ten in Canada by year, gender, age, and placement type; and 2) assess trends in the prevalence of out-of-home care over time by gender and placement type.

## Methods

### Data Source

The HBSC study is an international cross-sectional school-based survey conducted every 4 years by the World Health Organization (WHO). In Canada, Queen’s University and the Public Health Agency of Canada (PHAC) coordinate the survey. We used data from the 2002, 2006, 2010, 2014, and 2018 cycles. The self-report survey sampled school-aged children (who we also refer to as *students*) in grades six through ten attending public schools in all provinces and territories in Canada; Prince Edward Island and New Brunswick did not participate in the 2010 cycle, and Nunavut did not participate in 2018. The HBSC has a multi-stage cluster design whereby students are nested within classrooms, schools, school boards, and in provinces/territories [12]. The sampling approach treated provinces and territories as distinct strata and sampled each stratum through a method that accounted for school characteristics including language of instruction, public or separate (i.e., Catholic) designation where applicable, and community size. Because of mixed grade levels in some of the selected classrooms, the sample included some students in grade five and above grade ten [13]; this group accounted for a small percentage of respondents overall (0.9%, n = 791) from 2002 to 2018.

Some types of schools, such as those in specific Indigenous communities (i.e., First Nations reserves), private schools, and homeschooled children, were not included in the HBSC sample frame. Based on recent education data, the federal government estimated that 69% First Nations students living on reserve (n = 84,286) attended schools operated by Self-Governing First Nations and other First Nations in 2021 [14]. And in 2019/2020, fewer than 10% of students (n = 433,152) in Canada attended private/independent schools and 0.7% (n = 37,287) were home-schooled [15].

Classes within sampled schools had an approximately equal chance of study selection. Following consent procedures by each jurisdiction and school, parents/guardians/caregivers and students received consent forms [12]. All students in the selected classrooms were invited to complete the survey questionnaire. A combined total of 93,720 students anonymously completed the voluntary, self-reported survey, though the number of participants varied between cycles (Table 1). Surveys were completed by either by pen-and-paper or via a web-based program.

**TABLE 1:** Sample characteristics, Health Behaviour in School-aged Children study, Canada, 2002–2018.

Survey year	Students, n	Family structure question^a^				Proportion Female %	Age		
		Valid		Missing/invalid			Average (SD) years		Proportion 14+ years old, %
		n	%	n	%				
2002	7,234	7,183	99.3	51	0.7	53.6	13.7	(1.5)	41.8
2006	9,572	9,366	97.8	206	2.2	52.6	14	(1.5)	52.5
2010	25,838	24,729	95.7	1,109	4.3	50.6	13.8	(1.4)	47.2
2014	29,759	28,026	94.2	1,733	5.8	50.7	14	(1.6)	53.3
2018	21,317	20,952	98.3	365	1.7	50.5^b^	13.9	(1.3)	47.8
Total	93,720	90,256	96.3	3,464	3.7	51.5	13.9	(2.0)	49.4

SD, standard deviation.

^a^ For analysis of out-of-home care prevalence, this project included records with valid answers only (N = 90,256). It did not include those who did not answer or provided an invalid answer to the family structure question.

^b^ Does not include 2.0% of students who chose the option “neither applies to me” (before 2018, gender was measured as a dichotomous variable: male/female).

Source: Health Behaviour in School-aged Children (HBSC) study, 2002–2018 (N = 93,720).

The questionnaire was designed by WHO’s international HBSC project network to facilitate global comparisons of adolescent health and wellbeing [16]. Children were asked about their family structure, including whether they resided in a foster home or children’s home, with extended family, with parent(s), and/or with another person [16].

In Canada, the HBSC Study received ethics approval from research ethics boards at Queen’s University and PHAC/Health Canada [17]. Our analysis was exempt from research ethics board approval as per Canada’s *Tri-Council Policy Statement: Ethical Conduct for Research Involving Humans* because we used the data for public health surveillance [18].

### Variables

To answer the family structure question, students could choose any of up to eight options to identify who resided in the “home where [they] live all or most of the time” (e.g., “mother,” “father,” “stepmother,” “I live in a foster or children’s home”). In the 2002, 2006 and 2010 cycles, students were asked about their first and second homes. For consistency, with the 2014 and 2018 cycles which did not have information on a second home, we used only students’ first home responses. Supplementary Table S1 contains information on the questions and response options for each cycle.

From the content of the family structure question, we derived broad categories for two types of out-of-home care “placements,” as they are often called in the child welfare system [5, 19]. These categories were: 1) foster/children’s home (any association with foster/children’s home); 2) kinship home (living with a grandparent and/or a sibling without a parent or stepparent in the house); 3) living with parent or stepparent; or 4) missing/invalid. Foster/children’s home and kinship homes are the most common types of out-of-home placements in Canada (3, 11). However, in HBSC it is not possible to distinguish between a foster home and a children’s home (i.e., a group home) in the data due to the structure of the response options in the survey. Foster/children’s home and kinship home were aggregated for analysing determining the total prevalence of out-of-home care, which we reported as “All placements combined.” Multiple answers to the family structure question were allocated into one of these categories (Supplementary Table S2). We conducted a sensitivity analysis to assess the impact of including foster/children’s home respondents who still reported living with family members on the estimate of foster/children’s home prevalence (Supplementary Table S4).

We examined the point prevalence of out-of-home care by survey year, gender, and age group. For gender, the 2002 to 2014 HBSC questionnaires had binary response options: male or female. The 2018 cycle added “Neither term describes me” as a response option. Similar to previous studies, we refer to the latter category as non-binary gender [20, 21]. We categorized age into two groups: 13 or younger, and 14 or older.

### Data Analysis

We used R version 4.2.2 for the statistical analyses. The R survey package allowed for the clusters at the school-level to be specified for the 2010, 2014 and 2018 cycles [22]. For the 2002 and 2006 cycles, a weight of 1 was given to each respondent. We assumed independence among cycles since the inclusion probability of being selected in two cycles is virtually zero. We excluded respondents with missing responses to the age and gender questions (n = 1,208) from the dataset. Across the five cycles, the family structure question had on average 3.6% missing responses (ranging from 0.7% to 4.5%). Among the students who did not answer this question, a higher percentage were male (2.4% vs. 1.3%) and 13 and younger (2.2% vs. 1.5%); the differences were significant (p < 0.05 on chi-square tests).

To determine the prevalence of out-of-home care for each survey year, the numerator included students who responded affirmatively to a specific family structure category; the denominator comprised all valid responses to the family structure question. Prevalence estimates were stratified by year, gender, age group, and placement type. The use of sampling weights enabled the calculation of population estimates, as they adjusted for observed differences in the expected population by grade within each province and territory in the cycle [23].

To produce pooled prevalence estimates for the entire period, we used generic inverse variance pooling in the *metaprop* function from the R package “meta” where the prevalence estimates were logit-transformed (i.e., converting the proportions to log-odds). We examined trends in out-of-home care over time by regressing cycle against prevalence stratified by gender (overall, female and male) and age group (overall, 13 and younger, and 14 and older) using Joinpoint software (version 5.0.2, National Cancer Institute, Bethesda, MD, USA). We log-transformed the dependent variable in these regressions and assessed the statistical significance of the trend coefficient using a 95% confidence interval (CI).

## Results

The sample consisted of a total of 93,720 students who responded to the 2002, 2006, 2010, 2014, and 2018 questionnaires (Table 1). Overall, the average age of students was 13.9 years of age (SD = 2.0; mean age per cycle ranged from 13.7 to 14.0), and the proportion of female students was 51.5% (range: 50.5%–53.6%). An estimated 3.4% (95% CI: 3.3%–3.5%) of students reported living in out-of-home care (Table 2). The estimate of foster/children’s home prevalence was sensitive to the inclusion of foster/children’s home with family contact. The prevalence estimate decreased the most from the 2002 and 2018 cycles, from 0.9% to 0.3%, and 1.1%–0.5%, respectively. These were cycles where there were more foster/children’s home and family contact respondents (Supplementary Table S4).

**TABLE 2:** Pooled estimated prevalence of out-of-home care among children and youth by placement type, gender, and age group, Health Behaviour in School-aged Children study, Canada, 2002–2018.

Demographics	Prevalence % (95% Confidence Interval) by placement type		
	All placements combined	Foster/Children’s home	Kinship home
Total	3.5 (3.0, 3.9)	1.1 (0.8, 1.3)	2.4 (2.1, 2.6)
Gender			
Female	3.4 (3.0, 3.8)	1.0 (0.8, 1.2)	2.4 (2.2, 2.5)
Male	3.5 (3.0, 3.9)	1.2 (0.9, 1.4)	2.4 (2.1, 2.6)
Neither^b^	5.0 (1.0, 7.6)	1.8 (0.0, 3.1)	3.2 (1.0, 4.5)
Age group			
≤13 years	3.2 (2.7, 3.5)	1.0 (0.8, 1.2)	2.2 (2.0, 2.4)
≥14 years	3.8 (3.3, 4.1)	1.2 (0.9, 1.4)	2.6 (2.3, 2.7)

^a^ Does not include those with a missing/invalid answer to the family structure question (n = 3,464).

^b^ This category was available only in 2018 and comprised 2.0% of students, who chose the option “neither applies to me” (before 2018, gender was measured as a dichotomous variable: male/female).

Confidence Interval (CI), chi-square p = 0.05; no statistically significant differences were found at the p < 0.05 level.

Source: Health Behaviour in School-aged Children (HBSC) study, 2002–2018 (N = 90,256).

**FIGURE 1:**
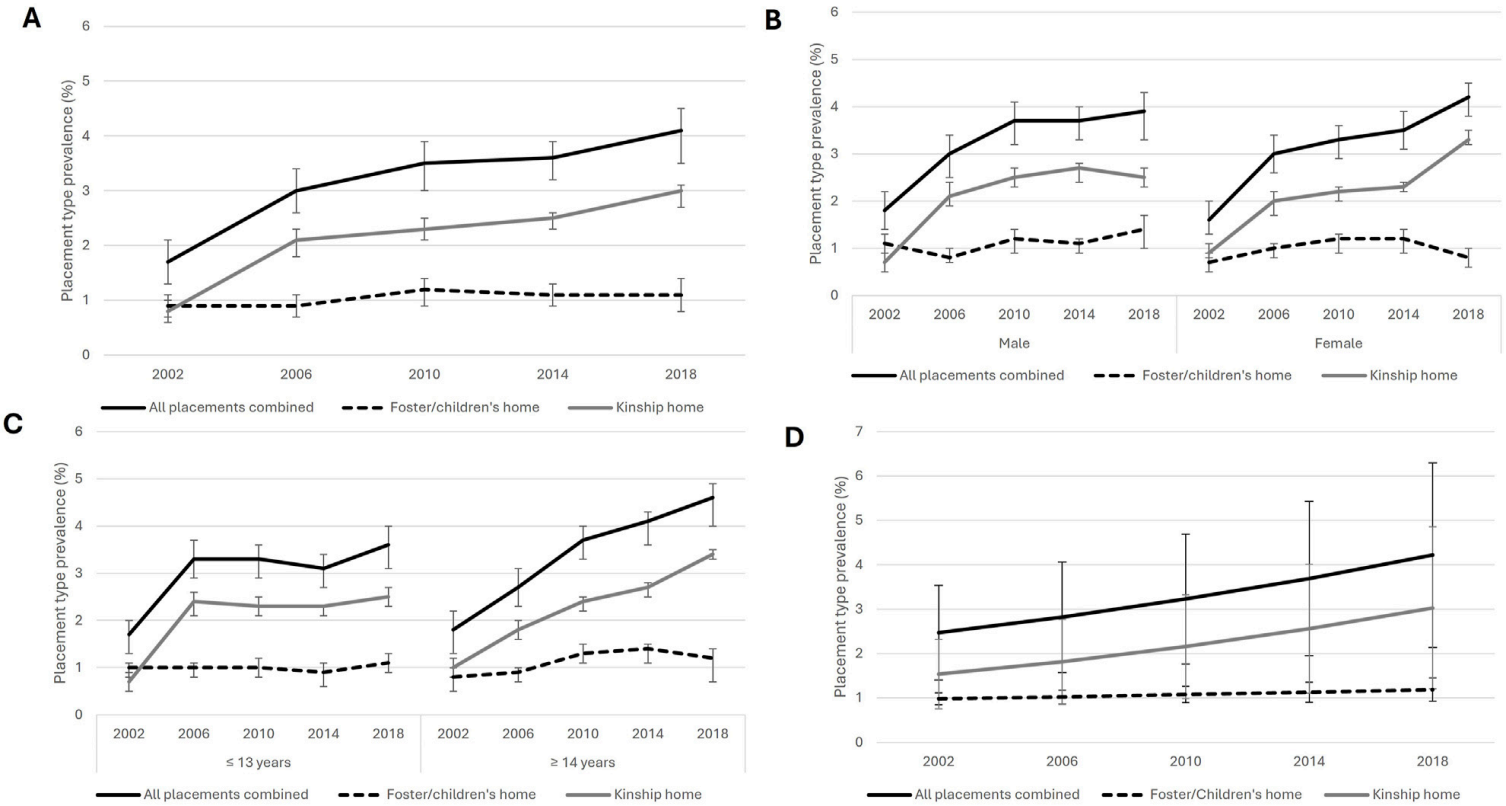
Estimated trends: **(A)** Overall prevalence of placement types, 2002 to 2018; **(B)** Prevalence of out-of-home care, by gender and placement type, 2002 to 2018; **(C)** Prevalence of out-of-home care, by age groups and placement type; **(D)** Modelled prevalence of placement types, 2002 to 2018 (Health Behaviour in School-aged Children study, Canada, 2002-2018).

More children and youth lived in a kinship home (2.4%, 95% CI: 2.3%–2.5%) than in a foster/children’s home (1.1%, 95% CI: 1.0%–1.1%) (Figure 1A). Overall, no significant differences emerged between the percentage of females and males in out-of-home care (Table 2; Figure 1B). In 2018, 5.0% (95% CI: 1.0%–7.6%) of non-binary students reported living in out-of-home care. At the p = 0.05 level (based on the chi-square test), there was no evidence indicating that the prevalence of out-of-home care differed between students aged 13 or younger (3.2%, 95% CI: 2.7%–3.5%) and 14 or older (3.8%, 95% CI: 3.3%–4.1%) (Table 2; Figure 1C).

Overall, the trend of students living in out-of-home care increased by 14.4% every cycle (95% CI: 3.9%–31.8%) (Table 3), that is, from 1.7% in 2002 to 4.0% in 2018 (Supplementary Table S3). The modelled prevalence is displayed in Figure 1D. Between 2002 and 2018, prevalence increased more for kinship homes, from 0.8% to 2.9% (increase of 2.1 points), which was an average of 18.5% (95% CI: -4.3%–61.2%) per cycle, compared with the prevalence of foster/children’s homes, which increased from 0.9% to 1.1% (increase of 0.2 points), which was an average of 5.0% increase per cycle (95% CI: -0.3%–12.4%). We did not observe gender differences (for males and females) in overall or placement type trends.

**TABLE 3:** Estimated trends in out-of-home care prevalence by placement type and gender, Health Behaviour in School-aged Children study, Canada, 2002–2018.

Gender	Average Percent Change per Cycle (95% CI) by placement type		
	All placements combined	Foster/Children’s home	Kinship home
Overall	14.4 (3.9, 31.8)	5.0 (−0.3, 12.4)	18.5 (−4.3, 61.2)
Female	16.6 (−0.7, 46.9)	1.2 (−26.2, 54.4)	25.1 (10.8, 49.9)
Male	11.6 (−0.5, 31.9)	8.3 (−2.0, 22.3)	11.1 (−4.9, 40.2)

Chi-square, p < 0.05; no statistically significant differences were found at the p < 0.05 level.

Source: Health Behaviour in School-aged Children (HBSC) study, 2002–2018 (N = 90,256).

## Discussion

From 2002 to 2018, 3.4% of students reported living in out-of-home care, with 1.1% in foster/children’s homes and 2.4% in kinship homes. The overall prevalence increased by 14.4% per cycle, with kinship homes rising at a faster rate (18.5% per cycle) than foster/children’s homes (5.0% per cycle). Our estimates of the prevalence of out-of-home care differed from those derived from other data sources. For instance, the 2018 HBSC estimate of 4.1% exceeds both the 2016 Census estimate of 1.4% (defined as children not living with their parents) [24] and the 2021 CCWIS estimate of ∼1% [5].

The CCWIS found that foster homes were the most common type of placement, whereas the HBSC reported more children in kinship homes than foster/children’s homes. This discrepancy is likely related to the broader definition of kinship home that we used with HBSC compared to the definition used in CCWIS. Our definition for HBSC data may have led to higher estimates of kinship prevalence since it did not necessarily require involvement with child welfare services, which was a criterion for inclusion in CCWIS data [5]. Despite differences in definitions, both the CCWIS and the HBSC data suggest an increasing trend in the proportion of kinship placements. This trend is consistent with policies that prioritize kinship placements over group home and foster care placements with non-relatives [25, 26].

Differing age coverage is an additional factor that may have contributed to discrepancies between estimates. Census-based analyses restricted the population to those aged 14 and under [7, 24, 27] even though the census itself enumerates children and youth in foster care up to age 24 [28]. By contrast, the HBSC study samples students in grades six through ten and typically does not include participants younger than 11. In CCWIS, children and youth up to the age of the majority (between 16 and 19 years) and beyond are included; based on data from 2021, 60% of children and youth in out-of-home care were younger than 12 [5].

Similar to recent analyses of the Census [7] and CCWIS [5], the percentage of females and males in out-of-home care were not significantly different in HBSC data. Furthermore, no gender differences were evident in the trends across placement types. In the 2018 HBSC, 0.8% and 1.4% of female and male students, respectively, reported being in foster homes, whereas 1.8% of non-binary students reported living in a foster/children’s home. These differences were not statistically significant, but our finding about non-binary students seems consistent with a study in a large Canadian province (British Columbia) which showed that non-binary children represented a greater proportion of respondents in placement than not in placement [29]. However, this result should be interpreted cautiously as the confidence interval is wide and based on a single year of data.

### Limitations

The HBSC’s representative sampling across Canadian provinces and territories is valuable for estimating the prevalence, trends, and demographics of self-reported out-of-home care among children and youth in grades six to ten. However, there are several methodological considerations when interpreting the results.

Our definition of kinship home was based on whether the respondent reported living with extended family without their parent(s) present—not necessarily with any child welfare involvement. This categorization likely does not correspond precisely with students placed outside their home through a judicial or administrative decision of a child welfare authority. However, a previous study from the province of Nova Scotia found that 80% of grandparents acting as primary caregivers for their grandchildren (i.e., kinship care) reported that they gained custody because of involvement with child welfare services [30]. If this were true nationally, which is unclear, it is possible that this methodological concern may have had only a small effect on the validity of kinship estimates from the HBSC.

The foster/children’s home response option on the HBSC family structure question does not differentiate between group homes with staff and ‘family-based’ foster homes. Based on administrative data, group homes or institutional care account for approximately 11.3% of placements in Canada [5]. HBSC data may appear to yield higher estimates of the prevalence of foster home placements compared with data sources that make this distinction.

Missing/invalid responses to the family structure question averaged 3.6%. Qualitative research suggests that children in out-of-home care may be reluctant to share information about their living situation to avoid stigmatization [31]. If non-response to the family structure question is positively correlated with being in a placement, HBSC data might underestimate the prevalence of out-of-home care.

### Directions for Future Research

Given the feasibility of using HBSC data to identify students in out-of-home care, the data may also be useful for better understanding the health status of this population. Although child welfare involvement is recognized as a determinant of health [3], national data sources are limited in their ability to comprehensively assess the health and wellbeing of children in out-of-home care [6], though some provincial data sources exist [29].

National surveys of children’s health include the Canadian Health Survey on Children and Youth (CHSCY) and the Cannabis, Obesity, Mental health, Physical activity, Alcohol, Smoking, and Sedentary behavior (COMPASS) Study. CHSCY is a nationally-representative population-based survey of children and youth aged 1 to 17 who are eligible for the Canada Child Benefit, a near-universal income support program. However, the Canada Child Benefit does not cover children who are under the custody or guardianship of a child welfare department or agency, which means that CHSCY excludes children in out-of-home care from its sampling frame [32]. Like the HBSC, COMPASS is a school-based health survey. However, it uses a convenience sample of secondary schools [33] and does not ask students explicitly about placement in out-of-home care [34]. Since HBSC includes and can identify children and youth in out-of-home care, and collects data about risk and protective factors and health status, it is uniquely positioned as a data source that can be used to strengthen the evidence about the health and wellbeing of a vulnerable population group. Notwithstanding the potential uses of HBSC data, there are also several possible ways the questionnaire could be revised to improve data on out-of-home care.

First, the HBSC questionnaire does not distinguish between foster homes and group homes. Group homes are a form of institutional care which create distinct risks for children [35]. Being able to further stratify data by specific placement type would help with both population surveillance and research on health outcomes. Second, the phrasing of the family structure question makes it difficult to compare HBSC estimates to point-in-time prevalence, which a common approach to data capture [4, 5]. Presently, the questionnaire asks who resides in “the home where you live all or most of the time.” This is a challenging time frame because it is an open and undefined period. Placement instability for those in out-of-home care [36] could impact the accuracy of responses. As an alternative, the question could be revised to specify a common period or reference date. Finally, in our sensitivity analysis we found that prevalence estimates were sensitive to the methodology for handling responses where students reported living in foster/children’s homes *and* had family contact. This group falls into a ‘grey area’ between “foster/children’s home” and “living with a parent or parents” due to the family structure response options. This suggests a need to further disaggregate these data.

### Conclusion

The prevalence of out-of-home care among school-aged children in Canada was higher that previous estimates based on census and administrative data. There did not appear to be significant differences between males and females or across age groups. From 2002 to 2018, the data showed a trend towards increased prevalence of kinship home placements, which were more common than foster/children’s home placements overall. Previous estimates with administrative data also found that kinship prevalence increased over a similar period [5], which suggests that this is a possibly convergent finding across data sources. Future studies with HBSC data may be useful for assessing risk and protective factors and health status among children and youth in out-of-home care, both in Canada and globally, especially if the family structure questions can be improved.
