# Supplementary material for: Prevalence of Out-Of-Home Care Among School-Aged Children in Canada, 2002–2018: An Analysis of Nationally-Representative Student Survey Data
Source: Int J Public Health. 2025 Nov 18;70:1608481. doi: 10.3389/ijph.2025.1608481 (PMC12669065; doi:10.3389/ijph.2025.1608481)
Supplement: Supplementary file 1 [file Supplementaryfile1.docx]

**Supplementary Tables**

Prevalence of out-of-home care among school-aged children in Canada, 2002-2018: an analysis of nationally-representative student survey data

**Supplementary Table S1**. Family Structure and Gender as defined in the Canadian Health Behaviour in School-aged Children (HBSC) Study, 2002-2018

| **Domain** |  | **HBSC Survey Year** | | |
| --- | --- | --- | --- | --- |
|  | **2002** | **2006 and 2010** | **2014** | **2018** |
| Family Structure | Now we’d like to ask you about who you live with. Not everyone lives with both their parents. Sometimes people live with just one parent, sometimes they have two homes or two families.  Please fill in column A for your main or your only home. Fill in column B if you have a second home (not including holiday or summer houses).  Please mark all the people who live here. Adults:   1. Mother 2. Father 3. Stepmother (or father’s girlfriend 4. Stepfather (or mother’s boyfriend) 5. Grandmother 6. Grandfather 7. I live in a foster or children’s home 8. Someone or somewhere else *(please write it down)* | All families are different (for example, not everyone lives with both their parents, sometimes people live with just one parent, or they have two homes or live with two families) and we would like to know about yours. Please answer this first question for the home where you live all or most of the time and tick the people who live there.  Do you have another home or another family, such as the case when your parents are separated or divorced? Please tick the people who live there:  *Potential responses*   1. Mother 2. Father 3. Stepmother (or father’s girlfriend) 4. Stepfather (or mother’s boyfriend) 5. Grandmother 6. Grandfather 7. I live in a foster or children’s home 8. Someone or somewhere else *(please write it down)* | Q48. All families are different (for example, not everyone lives with both their parents, sometimes people live with just one parent, or they have two homes or live with two families) and we would like to know about yours. Please answer this question for the home where you live all or most of the time and mark the people who live there.  *Potential responses*   1. Mother 2. Father 3. Stepmother (or father’s/mother’s girlfriend) 4. Stepfather (or mother’s/father’s boyfriend) 5. Grandmother 6. Grandfather 7. I live in a foster or children’s home 8. Someone or somewhere else *(please write it down)* | Q7. Please answer this question for the home where you live all or most of the time and mark the box beside the people who live there  *Potential responses*   1. Mother 2. Father 3. Stepmother (or father’s partner) 4. Stepfather (or mother’s partner) 5. Brother or stepbrother 6. Sister or stepsister 7. I live in a foster or children’s home 8. Someone else (e.g., grandparents). *Please write it down* |
| Gender | Q1. Are you male or female   1. Male 2. Female | Q1. Are you male or female?   1. Male 2. Female | Q4. Are you male or female?   1. Male 2. Female | Q4. Are you male or female?   1. Male 2. Female 3. Neither term describes me |

**Supplementary Table S2.** Classification of Student Responses to Family Structure Question, Canadian Health Behaviour in School-aged Children Study (HBSC), 2002-2018

|  | **Family Structure - Coarse** | **Family Structure - Granular** | **Unweighted count, n (%)** | **Student’s Response to Family Structure Question** (Y=Yes, N=No, N/A=Not applicable) | | | | | | | | |
| --- | --- | --- | --- | --- | --- | --- | --- | --- | --- | --- | --- | --- |
|  |  |  |  | Mother | Father | Stepmother | Stepfather | Brother | Sister | Grandparents (2002-2014) | Foster/children’s home | Someone else |
| All placements combined | Foster/children’s home | Foster/children’s home Exclusive | 690 (0.7) | N | N | N | N |  |  | N | **Y** | N OR N/A |
|  |  | Foster /children’s home + family contact | 269 (0.3) | **Y** OR **Y** OR **Y** OR **Y** | | | |  |  | **Y** | **Y** |  |
|  |  | Foster/children’s home + someone or somewhere else | 13  (0.0) | N | N | N | N |  |  | N | **Y** | **Y** (excluding grandparent in 2018^a^) |
|  | Kinship | Kinship grandparents | 1,331 (1.4) | N OR N/A | N | N | N |  |  | **Y** | N | **Y** (including grandparent in 2018^a^) |
|  |  | Kinship sibling | 206 (0.2) | N | N | N | N | **Y** OR **Y** | | N | N | N |
|  |  | Somewhere else exclusively | 1,174 (1.2) | N OR N/A | N OR N/A | N OR N/A | N or N/A |  | | N | N | **Y^b^** |
| Living with a parent or parents | Living with a parent or parents | Living with a parent or parents | 87,618 (92.3) | **Y** OR **Y** OR **Y** OR **Y** | | | |  |  |  | N | **Y** |
|  | Missing/invalid | No valid response to family structure | 3,326 (3.5) | N/A | N/A | N/A | N/A |  |  | N/A | N/A | N/A |
|  |  | No response to family structure Q | 301 (0.3) | N OR N/A | N OR N/A | N OR N/A | N OR N/A |  |  | N OR N/A | N OR N/A | N OR N/A |
|  | ^a^ In the 2018 HBSC, students were directed to indicate “someone else” if their grandparents lived in the home where they live all or most of the time. For this reason, for the foster + someone or somewhere else group in 2018, we excluded those respondents that selected someone else and listed grandparent. These were classified as kinship grandparents.  ^b^ Somewhere else exclusively is classified as kinship because free-text data from HBSC 2018 indicated that one third of these respondents were living with extended family (e.g., aunts, uncles, grandparents with aunt/uncle, cousin). | | | | | | | | | | | |

**Supplementary Table S3**. Estimated prevalence of out-of-home care types by gender and age group, Canada, 2002-2018 (N=90,256^a^)

| **Demographic variables** | **Placement type** | **Variable type** | **2002** | **2006** | **2010** | **2014** | **2018** | **Total** |
| --- | --- | --- | --- | --- | --- | --- | --- | --- |
|  |  |  | **Estimated prevalence (95% CI)** | | | | | |
|  |  |  |  |  |  |  |  |  |
| Gender | All out-of-home placement | Total | 1.7 (1.3–2.0) | 2.9 (2.5–3.3) | 3.4 (2.9–3.7) | 3.5 (3.1–3.8) | 4.0 (3.5–4.4) | 3.4 (3.3-3.5) |
|  |  | Female | 1.6 (1.3, 2.0) | 3.0 (2.6, 3.4) | 3.3 (2.9, 3.6) | 3.5 (3.1, 3.9) | 4.2 (3.8, 4.5) | 3.4 (3.0, 3.8) |
|  |  | Male | 1.8 (1.4, 2.2) | 3.0 (2.5, 3.4) | 3.7 (3.2, 4.1) | 3.7 (3.3, 4.0) | 3.9 (3.3, 4.3) | 3.5 (3.0, 3.9) |
|  |  | Neither | - | - | - | - | 5.0 (1.0, 7.6) | 5.0 (1.0, 7.6) |
|  | Foster/children's home | Total | 0.9 (0.7–1.1) | 0.9 (0.7–1.1) | 1.1 (0.9–1.3) | 1.1 (0.8–1.3) | 1.1 (0.8–1.3) | 1.1 (1.0-1.1) |
|  |  | Female | 0.7 (0.5, 0.9) | 1.0 (0.8, 1.1) | 1.2 (0.9, 1.3) | 1.2 (0.9, 1.4) | 0.8 (0.6, 1.0) | 1.0 (0.8, 1.2) |
|  |  | Male | 1.1 (0.9, 1.3) | 0.8 (0.7, 1.0) | 1.2 (0.9, 1.4) | 1.1 (0.9, 1.2) | 1.4 (1.0, 1.7) | 1.2 (0.9, 1.4) |
|  |  | Neither | - | - | - | - | 1.8 (0.0, 3.1) | 1.8 (0.0, 3.1) |
|  | Kinship | Total | 0.8 (0.6–1.0) | 2.0 (1.8–2.2) | 2.3 (2.0–2.4) | 2.4 (2.2–2.5) | 2.9 (2.7–3.0) | 2.4 (2.3-2.5) |
|  |  | Female | 0.9 (0.8, 1.1) | 2.0 (1.7, 2.2) | 2.2 (2.0, 2.3) | 2.3 (2.2, 2.4) | 3.3 (3.2, 3.5) | 2.4 (2.2, 2.5) |
|  |  | Male | 0.7 (0.5, 0.9) | 2.1 (1.9, 2.4) | 2.5 (2.3, 2.7) | 2.7 (2.4, 2.8) | 2.5 (2.3, 2.7) | 2.4 (2.1, 2.6) |
|  |  | Neither | - | - | - | - | 3.2 (1.0, 4.5) | 3.2 (1.0, 4.5) |
|  |  |  |  |  |  |  |  |  |
| Age Group | All out-of-home placement | Total | 1.7 (1.3–2.0) | 2.9 (2.5–3.3) | 3.4 (2.9–3.7) | 3.5 (3.1–3.8) | 4.0 (3.5–4.4) | 3.4 (3.3-3.5) |
|  |  | 13 and younger | 1.7 (1.3, 2.0) | 3.3 (2.9, 3.7) | 3.3 (2.9, 3.6) | 3.1 (2.7, 3.4) | 3.6 (3.1, 4.0) | 3.2 (2.7, 3.5) |
|  |  | 14+ | 1.8 (1.3, 2.2) | 2.7 (2.3, 3.1) | 3.7 (3.3, 4.0) | 4.1 (3.6, 4.3) | 4.6 (4.0, 4.9) | 3.8 (3.3, 4.1) |
|  | Foster/children's home | Total | 0.9 (0.7–1.1) | 0.9 (0.7–1.1) | 1.1 (0.9–1.3) | 1.1 (0.8–1.3) | 1.1 (0.8–1.3) | 1.1 (1.0-1.1) |
|  |  | 13 and younger | 1.0 (0.8, 1.1) | 1.0 (0.8, 1.1) | 1.0 (0.8, 1.2) | 0.9 (0.6, 1.1) | 1.1 (0.9, 1.3) | 1.0 (0.8, 1.2) |
|  |  | 14+ | 0.8 (0.5, 1.0) | 0.9 (0.7, 1.0) | 1.3 (1.1, 1.5) | 1.4 (1.1, 1.5) | 1.2 (0.7, 1.4) | 1.2 (0.9, 1.4) |
|  | Kinship | Total | 0.8 (0.6–1.0) | 2.0 (1.8–2.2) | 2.3 (2.0–2.4) | 2.4 (2.2–2.5) | 2.9 (2.7–3.0) | 2.4 (2.3-2.5) |
|  |  | 13 and younger | 0.7 (0.5, 0.9) | 2.4 (2.1, 2.6) | 2.3 (2.1, 2.5) | 2.3 (2.1, 2.3) | 2.5 (2.3, 2.7) | 2.2 (2.0, 2.4) |
|  |  | 14+ | 1.0 (0.8, 1.2) | 1.8 (1.6, 2.0) | 2.4 (2.2, 2.5) | 2.7 (2.5, 2.8) | 3.4 (3.3, 3.5) | 2.6 (2.3, 2.7) |

**Supplementary Table S4.** Sensitivity analysis of the impact of foster/children’s home and family contact counts on the estimate of foster/children’s home prevalence.

|  |  | Original analysis | | | Sensitivity analysis | | | | | |
| --- | --- | --- | --- | --- | --- | --- | --- | --- | --- | --- |
|  |  | Foster/Children’s home, inclusive of Foster/children’s home and family contact respondents | | | Foster/Children’s home, excluding foster/children’s home and family contact respondents | | | Foster/Children’s home, reclassified foster/children’s home and family contact as “Living with a parent or parents " | | |
| Year | Foster/Children’s home and family contact, unweighted n (%) | Foster/Children’s home, weighted n | Respondents, weighted n | Prevalence, % (95% CI) | Foster/Children’s home, weighted n | Respondents, weighted n | Prevalence, % (95% CI) | Foster/Children’s home, weighted n | Respondents, weighted n | Prevalence, % (95% CI) |
| 2002 | 39 (61.9%) | 63 | 7,183 | 0.9 (0.7, 1.1) | 24 | 7,144 | 0.3 (0.2, 0.5) | 24 | 7,183 | 0.3 (0.2, 0.4) |
| 2006 | 13 (15.1%) | 86 | 9,366 | 0.9 (0.7, 1.1) | 73 | 9,353 | 0.8 (0.6, 0.9) | 73 | 9,366 | 0.9 (0.7, 1.0) |
| 2010 | 69 (22.8%) | 293 | 24,953 | 1.2 (0.9, 1.4) | 214 | 24,874 | 0.9 (0.7, 1.0) | 214 | 24,952 | 0.9 (0.6, 1.1) |
| 2014 | 58 (20.4%) | 324 | 28,420 | 1.1 (0.9, 1.3) | 250 | 28,346 | 0.9 (0.8, 1.4) | 250 | 28,419 | 0.9 (0.6, 1.1) |
| 2018 | 86 (39.1%) | 234 | 20,986 | 1.1 (0.8, 1.4) | 99 | 20,851 | 0.5 (0.3, 0.6) | 99 | 20,985 | 0.5 (0.3, 0.6) |
| **Total** | 265 (27.7%) | 956 | 90,908 | 1.1 (0.8, 1.3) | 660 | 90,568 | 0.7 (0.5, 0.9) | 660 | 90,905 | 0.7 (0.5, 0.9) |
